# Avian extremity reconstruction via osseointegrated leg-prosthesis for intuitive embodiment

**DOI:** 10.1038/s41598-021-90048-2

**Published:** 2021-06-11

**Authors:** Sarah Hochgeschurz, Konstantin D. Bergmeister, Rickard Brånemark, Martin Aman, Attillio Rocchi, Flavia Restitutti, Michaela Gumpenberger, Matthias E. Sporer, Clemens Gstoettner, Anne-Margarete Kramer, Susanna Lang, Bruno K. Podesser, Oskar C. Aszmann

**Affiliations:** 1 Service for Birds and Reptiles, Department for Companion Animals and Horses, University of Veterinary Medicine Vienna, Vienna, Austria; 2 Clinical Laboratory for Bionic Extremity Reconstruction, Department of Surgery, Medical University of Vienna, Vienna, Austria; 3 Department of Plastic, Reconstructive and Aesthetic Surgery, University Hospital St. Poelten, St. Poelten, Austria; 4 Department of Orthopaedics, Gothenburg University, Gothenburg, Sweden; 5 Biomechatronics Group, MIT Media Lab, Massachusetts Institute of Technology, Cambridge, MA USA; 6 Center for Biomedical Research, Medical University of Vienna, Vienna, Austria; 7 Department of Anaesthesiology and Perioperative Intensive-Care Medicine, University of Veterinary Medicine Vienna, Vienna, Austria; 8 Diagnostic Imaging, Department for Companion Animals and Horses, University of Veterinary Medicine, Vienna, Austria; 9 Clinical Institute of Pathology, Medical University of Vienna, Vienna, Austria; 10 Division of Plastic and Reconstructive Surgery, Department of Surgery, Medical University of Vienna, Vienna, Austria

**Keywords:** Diseases, Trauma

## Abstract

For large avians such as vultures, limb loss leads to loss of ambulation and eventually death from malnutrition. Prosthetic devices may replace the limb, however, conventional prosthetic sockets are not feasible in feathered limbs and the extreme stress and strain of unreflected daily use in animals. Osseointegration is a novel technique, where external prosthetic parts are connected directly to a bone anchor to provide a solid skeletal-attachment. This concept provides a high degree of embodiment since osseoperception will provide direct intuitive feedback allowing natural use of the limb in gait and feeding. Here we demonstrate for the first time an osseointegrated bionic reconstruction of a limb in a vulture after a tarsometatarsal amputation with a longterm follow-up.

## Introduction

**Figure 1:**
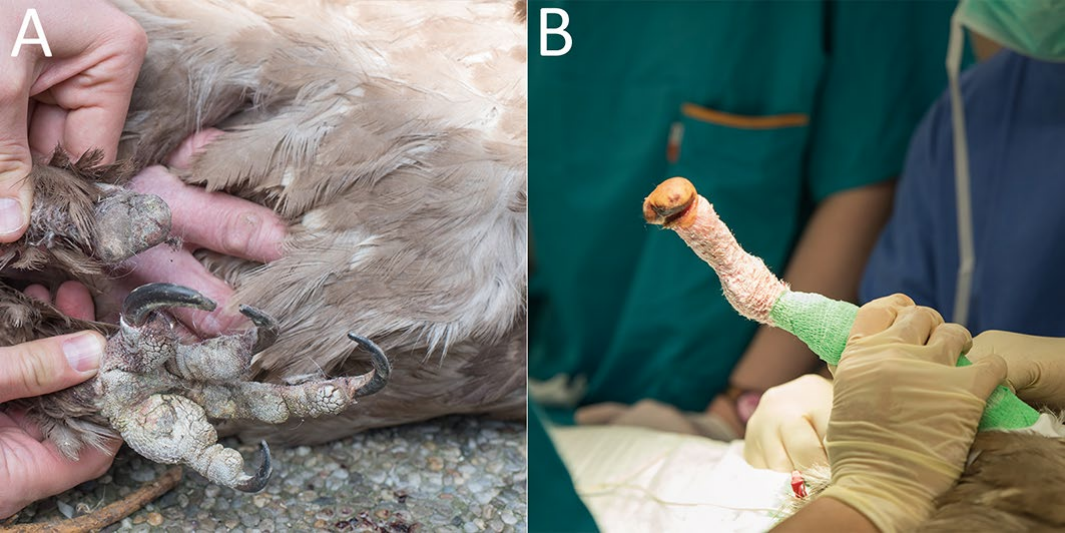
Amputation stump prior to surgery in a bearded vulture. (**A**) Shown is the intact left limb compared to the self-amputated right limb. (**B**) A large formation of granulation scar is visible at the distal end of the amputated limb with chronic ulceration due to heavy load during daily activities.

A one-year-old female bearded vulture (Gypaetus barbatus) with an amputation at the tarso-metatarsal level due to a strangulation injury to the right foot was presented to the Laboratory of Bionic Extremity Reconstruction at the Medical University of Vienna by the Service for birds and reptiles of the University of Veterinary Medicine Vienna. The bird suffered the amputation due to sheep wool used for nest building, which became entangled around the birds right foot and subsequently led to ischemia and necrosis of all toes at the distal tarsometatarsus. Prior to our consultation, the bird had received conservative care at the Richard Faust Breeding Centre near Vienna, where an increasing lameness with extensive muscle atrophy and chronic ulceration became evident in the right leg and it was observed that during landing maneuvers, she repetitively injured her stump skin (Fig. 1)^1^. In addition, the asymmetrical weight distribution led to the development of pododermatitis and hyperkeratosis on the contralateral left foot^2,3^.

As chronic ulceration further complicated the wound with progressive infection of the deeper tissues and bone^4^ and the animal suffered a general decline in overall health and weight, the decision was made to intervene surgically via an experimental osseointegration procedure to provide a prosthetic solution. Alternatively, the bird would have had to be euthanized due to a lack of treatment options in light of the rapid decline in health. Limb loss for any bird of prey or vulture would be lethal in the wild, firstly because they can’t feed themselves, but secondly because they would develop pododermatitis on the intact foot. Those individuals are also not able to reproduce.

Here, we describe the first application of the concept of bionic reconstruction including osseointegration in a bird.

## Methods

### Experimental design

The experimental treatment of the bird was with consent of the animal’s owner for primary curative purpose and to prevent the euthanasia in light of the rapidly declining health. According to Austrian legacy (TVG 2012) no ethical vote was therefore necessary.

**Figure 2:**
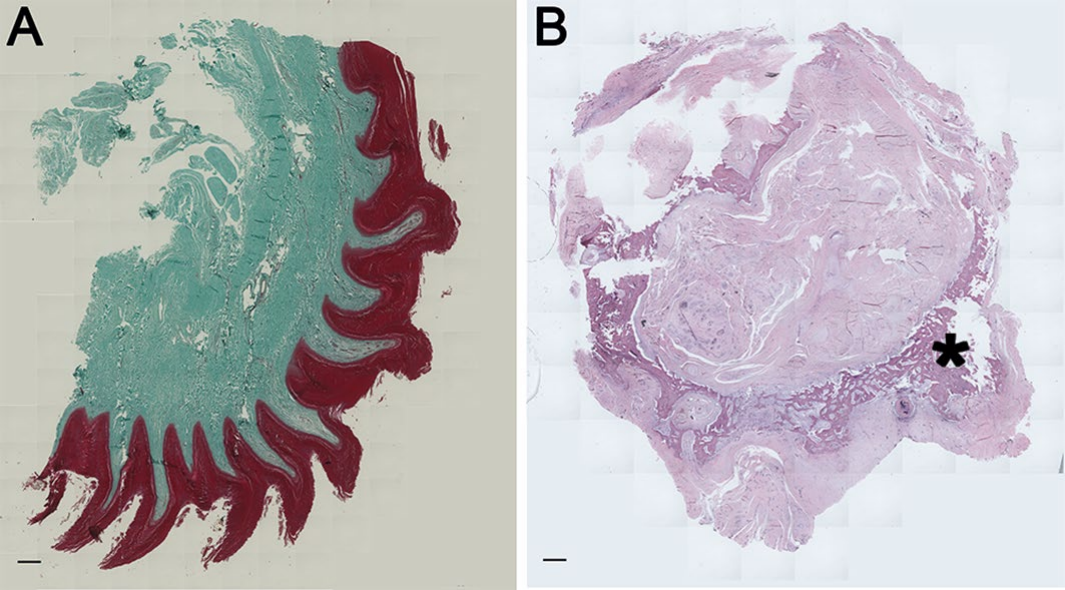
(**A**) Masson Trichrome staining of a stump skin biopsy: Shown is the specific skin architecture of the vulture's stump in a cross-section. Red indicates the epithelium and cyan the subcutaneous tissue. The osseointegration device is surrounded by this tissue and provides a safe enclosure of the skin penetrating element. (**B**) HE-Staining of the tarsometatarsus bone showing cortical bone (see asterisk) filled with connective tissue and cartilage, surrounded by thin cortical bone in a cross-section. Bar indicates 1 mm.

To prevent the need for euthanasia of the bird, a viable long-term prosthetic solution had to be designed, based on osseointegration of the prosthesis. In order to apply the concept of osseointegration to this particular species several preconditions had to be ascertained. Firstly, the remaining length of bone and geometry of intraosseous medullary cavity had to be analyzed to assure that a titanium implant of sufficient length and diameter could be designed for adequate stability of the external prosthesis. Secondly, the skin overlying the percutaneous port must be sufficiently vascularized and supple with no skin appendages to allow surgical manipulation and subsequent healing (Fig. 2A). Thirdly, bone physiology must be conductive of osseointegration and allow quick integration of a titanium implant.

### Imaging

To address the above issues, a computed tomographic (CT) scan was performed with a 16-slice helical CT (Siemens Somatom Emotion, Vienna, Austria), using 80 mAs, 130 kV, rotation time 1.5 s, pitch 0.8 and slice thickness 0.75 mm. The scan was reformatted with an ultra-sharp kernel for bony window, with FOV 55 × 55 mm, matrix size 512 × 512 and increment 0.6 mm. The CT scans allowed to evaluate and measure the stump and the overall length of the remaining tarsometatarsal bone as well as the internal diameter of the medullary cavity. The primary measurements were approximately 5 cm of length; however, the intramedullary cavity did only provide a 4 cm distance of parallel cortical bone of adequate geometry to house a circular implant design (Fig. 2B).

### Anatomical and physiological studies

Additionally, we conducted anatomical studies on deceased vulture cadavers to investigate the surgical anatomy and ex-vivo evaluations to test the feasibility of this novel approach. In the deceased specimens, two important details could be observed. First, the stump coverage requires a dorsomedial pedicled fasciocutaneous flap, as this flap carries the necessary vascular pedicle based on a medial artery and a dorsal vein. Second, the extremity and specifically the bone anatomy of vultures being scavengers, differs from many other birds that hunt on prey. Hunting birds need very strong finger flexors to catch and hold their prey. Therefore, they are equipped with long tendons that are closely attached to the tarsometatarsal bones to form a hypomochlion and exert tremendous strength to the individual claws when extending the phalanges. Consequently, the tarsometatarsal bone is U-shaped, to form an osseofibrous tunnel to house the tendons. As vultures do not hunt but feed on cadavers, their claws do not require being as strong and thus the tendons do not need such strong support. As a consequence, their distal tarsometatarsal bone is almost round and amenable for a circular intramedullary implant. These bones consist of a collagen matrix including type I, IV and XII, which is packed with hydroxyapatite microcrystals for additional strength while reducing overall weight (Fig. 2B)^5^. Additionally the weight to stability ratio is improved by a fused tarsometatarsus, without the typical joints in-between, comprising of the distal tarsal bones and metatarsals II, III and IV^6^. A further distinction to humans and other species is that bone healing occurs rapidly in birds, with fractures being stable after 3–4 weeks, which is an advantage for bone surgery in these animals^6,7^.

### Surgery

**Figure 3:**
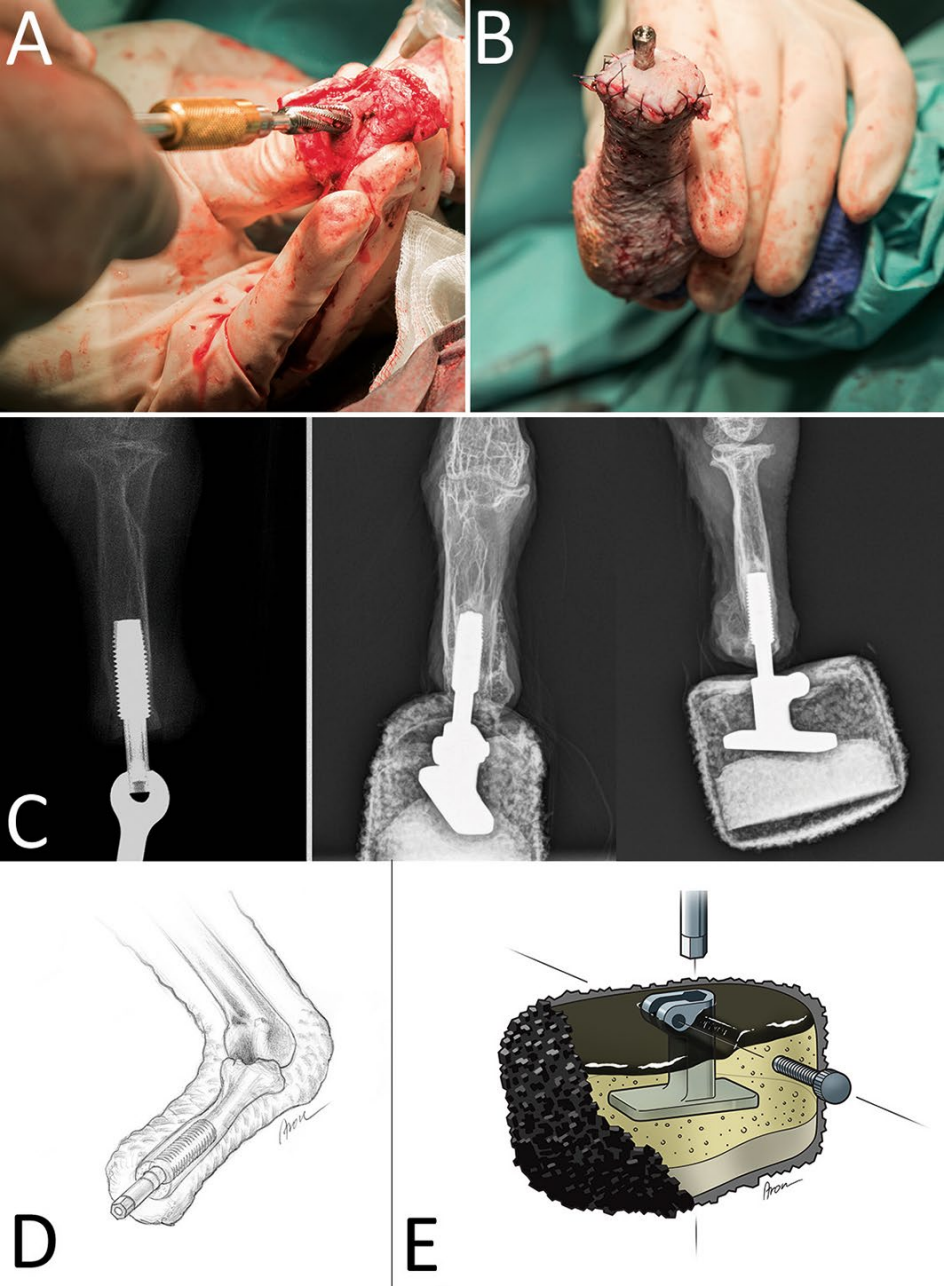
Concept of osseointegration in the vulture. (**A**) Implantation of the osseoimplant after osteotomy with a specialized drilling device. (**B**) The revised stump with osseoimplant piercing through a skin opening after wound closure. Copyright by Felix Gantenbein. (**C**) Radiographs of the implant during surgery (left, dorsoplantar view), and after the surgery with the mounted prosthesis (middle/dorsoplantar view and right/mediolateral view). (**D**) Schematic drawing of the osseoimplant after surgery, located in the medullary cavity. (**E**) Design of the prosthetic attachment to the bone implant. Various layers of cohesive soft materials are located around the central metal attachment to the prosthesis. On the prosthesis’ surface a rough rubber surface provides traction and stability.

After inducing general anesthesia (for further anesthesia details see Supplementary methods), the feathers were plucked, and the surgical side was aseptically prepared. A tourniquet was placed around the extremity and a lateral approach to the tarsometatarsus with a semicircular skin incision was made as described in previous protocols in other species^6^. Great care was taken not to injure the medial metatarsal vein as well as the cranial metatarsal artery to have sufficient blood supply for the flap covering the stump^6^. A transversal osteotomy was performed 2.8 mm proximal to the distal end of the bone to open the medullary canal using a specifically designed instrument for the osseo-implant (Integrum, Sweden; Fig. 3). Then the threaded titanium fixture was inserted longitudinally into the medullary canal. Operative success and positioning were confirmed by intraoperative fluoroscopy (Siemens Cios Alpha, Germany). The stump was subsequently closed with a dorsomedial skin flap using single interrupted sutures and a 3 mm biopsy punch generated an opening for the prosthesis attachment (Fig. 3). Sterile gauze was wrapped around the abutment screw with additional bandage padding. For post-operative care, the bird received antibiotics (Enrofloxacin 10 mg/kg i.m.) for three weeks and analgesics (Buprenorphine 0.15 mg/kg i.m.) as well as anti-inflammatory drugs (Meloxicam 1 mg/kg, i.m.) for five days.

### Prosthetic design

**Figure 4:**
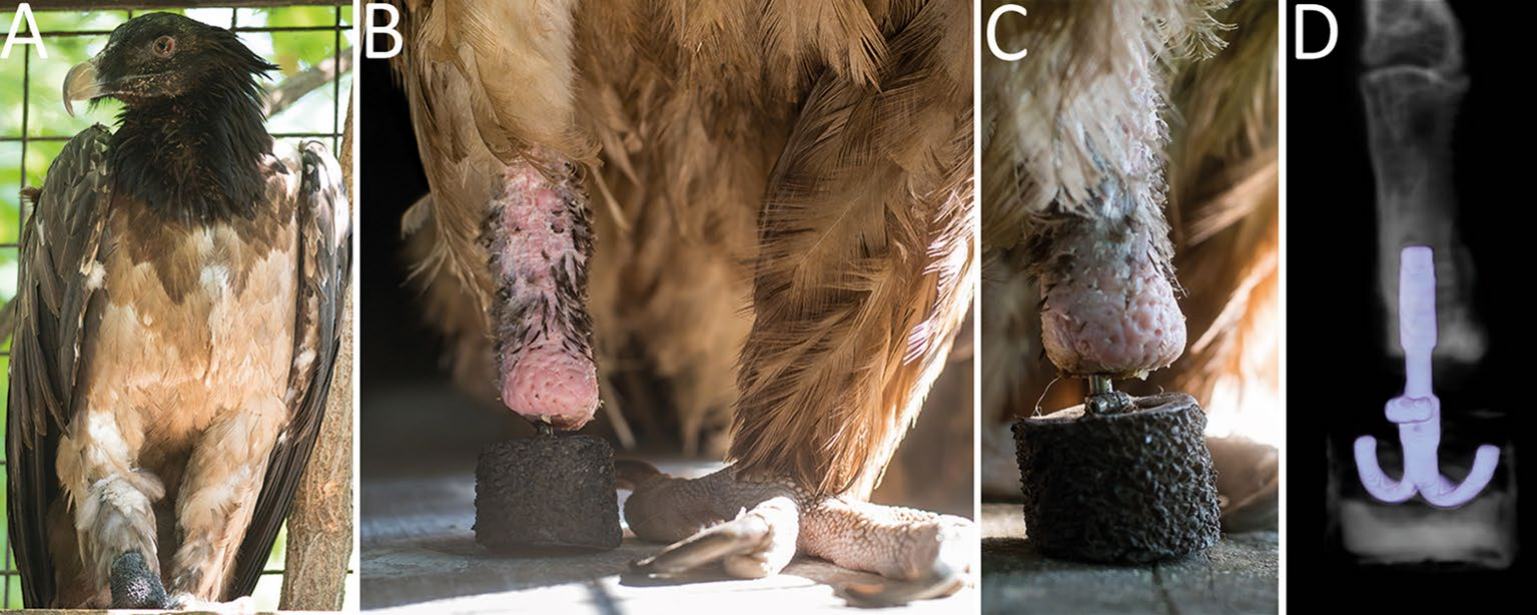
Postoperative images of the osseointegrated prosthesis. (**A**) The vulture shows a restored ability to stand and distribute weight on both extremities, preventing pressure-induced issues on the healthy limb. (**B**) Front view: The size and weight of the prosthesis is matched to the regular limb. Although the limb replacement does not contain the ability to grasp prey, the ability to maintain stance enables the bird to use the intact limb safely. (**C**) Side view: The prosthesis provides an irregular surface with traction and a shock-absorbing function by a specialized combination of materials for endurance and compressibility. Supplemental videos show the prosthesis in use during stance, gait and feeding. (**D**) A transparent osseous 3D model of the CT images demonstrates the situs and configuration of the implant.

For the design of the prosthesis no previous templates existed, and thus the specific needs were investigated based on anatomical dissections and functional analyses on living specimen. These indicated that vultures have an anisodactyl foot configuration, where one toe is located in the back and three in the front for gripping carrion or prey and being able to tear it apart with their beak^5^. In addition, the lower limb’s skin consists of tough scales and papilla adapted to the heavy use of these claws to prevent injuries and act as a protective layer against environmental stress^8^. Based on these specific requirements, we designed a durable, dirt-repellent and waterproof prosthesis, with shock absorbing properties for landing maneuvers with weight peaks manyfold of the typical 4–7 kg tare weight^2^. To match the properties of the intact limb, the length of the exoprosthesis measured a total of 35 mm in height with a weight of just 72 g to minimize unphysiological effects on flight and walking properties. Furthermore, the prosthesis was shaped cylindrically with a diameter of 30 mm to prevent peak rotational forces on the implant until it was ensured that it had fully integrated into the bone (Fig. 4).

### Postoperative prosthesis fitting

The bird was restricted from full weight bearing for three weeks and a follow-up CT scan showed adequate osseointegration at five weeks postoperatively (Fig. 4D). After three weeks, the prosthesis was attached for the first time. At six weeks, the initial lameness score improved from the 3/5 pre-operatively to 0/5 (0 = no lameness, 5 = no gait possible), showing rapid intuitive use of the specifically designed prosthesis. After three months the design was changed from the previous peg-leg to a more physiological shape. This prosthesis was constructed around a central titanium framework with 1.5 cm Silicone on the bottom and hard foam acting as a superficial layer (Fig. 4D). The attachement-system uses a screw to connect to the implant.

## Discussion

Embodiment describes the ability of intuitive use and natural integration of a prosthetic replacement and is a key element in successful restoration of extremity function^9,10^. Modern prosthetic extremity replacement has sparked much research during the past decades and provides human amputees with meaningful aids, but due to unintuitive control they remain behind the dexterity of their natural counterparts^11^. Therefore, they require active thought to realize an intended movement and visual control if the desired motion is to be adequately performed due to a lack of sensory feedback. Unlike natural motion, where movement sequences are rapidly learned and conducted without much thought, prosthetic motion therefore suffers from slow and unintuitive processing that in many cases leads to device abandonment^12,13^. These challenges become even more evident in animals, as they are not able to cognitively perceive and embody conventional prostheses as an extremity replacement and thus rapidly fail due to unintended use and material overload. These devices must thus be extremely durable and integrated into the animal’s body scheme in a very robust and reliable way in order to be beneficial for the most basic needs of the animal, such as ambulating, feeding and grooming in daily life. Animals consequently are very quick to dismantle and discharge non-intuitive devices and lack of solid embodiment will become evident immanently.

In recent years it has become increasingly evident, that embodiment is dependent on precise and reliable skeletal control which is best realized with direct skeletal attachment, a concept termed osseointegration^14^. In this procedure, a titanium-shaft for the attachment of the prosthesis is implanted into a bone’s medullar cavity of the remaining osseous structure in the stump. Thereby, a solid skeletal attachment is generated that eludes the problems of conventional sockets such as skin irritations, unstable attachment due to sweating or volume changes, and restricted range of motion^15,16^. This procedure has been employed in a limited number of patients throughout the last 30 years and yielded significant improvements with regard to intuitive prosthetic use and better embodiment^14–16^. Furthermore, the direct and solid skeletal connection offers these patients a direct sensory feedback termed osseoperception^14,15^. Despite the obvious risks of a bone-anchored implant that pierces the skin, infection rates are reasonably low, and patients adhere to the specific care instructions in order to maintain their additional performance provided by osseointegration^14–16^.

Traditionally, prostheses have been attached in humans to an amputation stump by socket-suspended-shafts, which are held to the limb by firm grip to the skin^17^. While this attachment is the standard of care, it requires regular adaption to the changing anatomy of the stump and still may be prone to motion during heavy load or sweating. Due to the bird’s limb anatomy with an irregular surface and feathers of bearded vultures, which cover even the distal aspect of the limb, this approach was not considered feasible. Socket-type prosthesis were previously used in a bald eagle (Haliaeetus leucocephalus), a red-lored Amazon parrot (Amazona autumnalis) with very promising results, but because of the wide variety of bird species, this approach can only be applied to certain species, not bearded vultures^18,19^. Furthermore, daily handling procedures of primarily wild-living birds cause stress and subsequently potentially life-threatening infections with aspergillus. These risks and furthermore the heavy load on a lower leg bird prosthesis and subsequent risk for pressure sores and skin infection, obstruct this application. Therefore a permanent solution which does not require donning and doffing was necessary^8^.

Osseointegration has significantly advanced prosthetic attachment in humans and thus seemed a unique possibility in an avian application due to a lack of alternatives^14,15^. Although, dogs and cats have successfully been treated with very promising results^20^, there is only one report of a white-naped crane (Grus vipio) where an osseointegrated implant was used, but lead to a avascular necrosis of the proximal tibiotarsus and subsequently to the euthanasia of the patient^21^. Our case report shows for the first time, the use of an osseointegrated implant in a bird patient with a successful outcome.

In this particular avian patient, osseointegration allowed the fitting of a coated titanium implant into the bone, whose size was determined by a CT scan. A custom-designed implant was made by Integrum Inc. (Mölndal, Sweden), with dimensions of 7 mm by 20 mm. A challenging aspect of avian surgery is anesthesia, as birds have a higher mortality rate (around 1.76%) compared to humans (0.02–0.05%), dogs (0.17%) and cats (0.24%)^22^. Especially blood loss and overall anesthesia time are major concerns in birds, which thus require meticulous planning for such an experimental procedure. Therefore, allogen blood was matched and collected from another vulture for potential transfusion.

With the final design, the bearded vulture was able to perform regular gait and flight maneuvers, showing a rapid and intuitive integration of the prosthesis into daily activities (Fig. 4). Although its grasping abilities are restricted to the intact left foot, the prosthesis is well-used to keep balance during feeding and beak-activities (see Supplementary Video S1). Over the past 18 months, observations of the bird in the aviary showed a completely normal behavior and excellent weight bearing on its right leg during gait and landing and usage of the prosthesis in grooming, to pin down food items and scratch herself. The bird does not show any physical limitations or pain and has regained initially lost weight as an indicator for overall well-being. While one may question the stability of such implants and fear the possibility of skin or bone infection, none of these problems have developed in our patient. Once, the bird required the fitting of a new connector between implant and prosthesis, which was conducted in anesthesia to minimize stress, analyze and minimally revise the stump morphology. Both clinical observations indicated an intact skin-penetration site and radiographs confirmed good osseointegration without any signs of osteomyelitis or loosening. This anecdotal case demonstrates how osseointegrated prosthetics can enable intuitive limb replacement in avian patients and prevent secondary morbidity or mortality.

## Data availability

All data is available on request with the authors.

## Supplementary Information

Supplementary Information 1.

Supplementary Video 1.
