# Author Correction: Avian extremity reconstruction via osseointegrated leg-prosthesis for intuitive embodiment

**DOI:** 10.1038/s41598-021-99053-x

**Published:** 2021-09-22

**Authors:** Sarah Hochgeschurz, Konstantin D. Bergmeister, Rickard Brånemark, Martin Aman, Attillio Rocchi, Flavia Restitutti, Michaela Gumpenberger, Matthias E. Sporer, Clemens Gstoettner, Anne-Margarete Kramer, Susanna Lang, Bruno K. Podesser, Oskar C. Aszmann

**Affiliations:** 1 Service for Birds and Reptiles, Department for Companion Animals and Horses, University of Veterinary Medicine Vienna, Vienna, Austria; 2 Clinical Laboratory for Bionic Extremity Reconstruction, Department of Surgery, Medical University of Vienna, Vienna, Austria; 3 Department of Plastic, Reconstructive and Aesthetic Surgery, University Hospital St. Poelten, St. Poelten, Austria; 4 Department of Orthopaedics, Gothenburg University, Gothenburg, Sweden; 5 Biomechatronics Group, MIT Media Lab, Massachusetts Institute of Technology, Cambridge, MA USA; 6 Center for Biomedical Research, Medical University of Vienna, Vienna, Austria; 7 Department of Anaesthesiology and Perioperative Intensive-Care Medicine, University of Veterinary Medicine Vienna, Vienna, Austria; 8 Diagnostic Imaging, Department for Companion Animals and Horses, University of Veterinary Medicine, Vienna, Austria; 9 Clinical Institute of Pathology, Medical University of Vienna, Vienna, Austria; 10 Division of Plastic and Reconstructive Surgery, Department of Surgery, Medical University of Vienna, Vienna, Austria

Correction to: *Scientific Reports* 10.1038/s41598-021-90048-2, published online 11 June 2021

The original version of this Article contained an error in the legend of Figure 3, where

“Concept of osseointegration in the vulture. (**A**) Implantation of the osseoimplant after osteotomy with a specialized drilling device. (**B**) The revised stump with osseoimplant piercing through a skin opening after wound closure.”

now reads:

“Concept of osseointegration in the vulture. (**A**) Implantation of the osseoimplant after osteotomy with a specialized drilling device. (**B**) The revised stump with osseoimplant piercing through a skin opening after wound closure. Copyright by Felix Gantenbein.”

The original Article has been corrected.

