# Supplementary material for: Avian extremity reconstruction via osseointegrated leg-prosthesis for intuitive embodiment
Source: Sci Rep. 2021 Jun 11;11:12360. doi: 10.1038/s41598-021-90048-2 (PMC8195993; doi:10.1038/s41598-021-90048-2)
Supplement: Supplementary file 1 — Supplementary Information 1. [file 41598_2021_90048_MOESM1_ESM.docx]

Title: Avian extremity reconstruction via osseointegrated leg-prosthesis for intuitive embodiment

**Authors:** Sarah Hochgeschurz¹*^§^, Konstantin D. Bergmeister^2,3§^**, Rickard Brånemark^4,5^ , Martin Aman^2,6^,** Attillio Rocchi**^7^, Flavia Restitutti^7^, Michaela Gumpenberger^8^, Matthias E. Sporer^2,6^, Clemens Gstoettner^2,6^, Anne-Margarete Kramer^6^, Susanna Lang^9^, Bruno K. Podesser^6^, Oskar C. Aszmann^2,10^**

**Affiliations:**

¹ Service for birds and reptiles, Department for Companion Animals and Horses, University of Veterinary Medicine Vienna, Austria

² Clinical Laboratory for Bionic Extremity Reconstruction, Department of Surgery, Medical University of Vienna, Austria

^3^ Department of Plastic, Reconstructive and Aesthetic Surgery, University Hospital St. Poelten, Austria

^4^ Department of Orthopaedics, Gothenburg University, Gothenburg, Sweden

^5^ Biomechatronics Group, MIT Media Lab, Massachusetts Institute of Technology, Cambridge, MA, USA

^6^ Center for Biomedical Research, Medical University of Vienna, Vienna, Austria

^7^ Department of Anaesthesiology and Perioperative Intensive-Care Medicine, University of Veterinary Medicine Vienna, Austria

^8^ Diagnostic Imaging, Department for Companion Animals and Horses, University of Veterinary Medicine, Vienna, Austria

^9^ Clinical Institute of Pathology, Medical University of Vienna, Vienna, Austria.

^10^ Division of Plastic and Reconstructive Surgery, Department of Surgery, Medical University of Vienna, Vienna, Austria

§ both Authors contributed equally

^*^Correspondence to:

[avianprosthetics@gmail.com](mailto:avianprosthetics@gmail.com)

Supplementary Materials:

**Supplementary Methods:**

**Anaesthesia:**

The vulture was physically restrained to inject Butorphanol (0.5 mg/kg) and Midazolam (1mg/kg) in the pectoral muscle. The sedation was sufficient to insert a 20-gauge catheter in the left basilic vein. General anaesthesia was subsequentially induced with Alfaxalon (2 mg/kg i.v.). Orotracheal intubation was achieved with a 5.0 mm Cole’s tube. General anaesthesia was maintained with Isoflurane in 25% oxygen administered via a non-rebreathing circle. Monitoring consisted of ECG, pulse-oximetry, side stream capnography, oesophageal temperature, and arterial-doppler blood pressure. The bird was warmed with a warm air blanket and received continuous balanced crystalloid solutions i.v. (5 ml/kg/h). The patient was mechanically ventilated with a pressure-limited modality, using a peak inspiratory pressure of 6 cmH_2_O, while respiratory rate was adjusted to maintain normocapnia. A combined sciatic-femoral nerve block was performed with Ropivacaine (2 mg/kg) using a nerve locator ^21^ and augmented with fentanyl infusions (0.02 mg/kg/h). The total anaesthesia time was 135 minutes, with a quick and uneventful recovery after Flumazenil application (0.1 mg/kg Intra nasally). Postoperative analgesia consisted of Meloxicam (1mg/Kg i.m.) and Buprenorphine (3µg/Kg i.m.)

**Materials and Methods**

Movie S1: The patient after successful surgery, fitted with the osseointegrated prosthesis. Shown are typical gait, stance and feeding behaviors of the bird, indicating a well-integrated use of the device into body image
